# Three-Dimensional Finite Element Analysis of Stress Distribution in Dental Implant Prosthesis and Surrounding Bone Using PEEK Abutments

**DOI:** 10.3390/biomimetics9080472

**Published:** 2024-08-03

**Authors:** Min-Ho Hong, Hyunsuk Choi

**Affiliations:** 1 Department of Dental Laboratory Science, College of Health Sciences, Catholic University of Pusan, Busan 46252, Republic of Korea; mhhong@cup.ac.kr; 2 Department of Dentistry and Prosthodontics, Daegu Catholic University School of Medicine, Daegu 42472, Republic of Korea

**Keywords:** dental implants, finite element analysis, PEEK, implant prosthesis

## Abstract

(1) Background: Polyetheretherketone (PEEK) has been used as an alternative to titanium in implant prosthetic systems, but its impact on stress distribution in implant systems needs to be investigated. This study aimed to compare the effect of polyetheretherketone (PEEK) and titanium abutments on implant prosthetic systems and the supporting bone using three-dimensional finite element analysis (FEA). (2) Methods: Three-dimensional finite element analysis was conducted using CATIA V5 and Abaqus V6.12 software to model mandibular first-molar implant systems with titanium and PEEK abutments. Under external loading conditions, finite element analysis was conducted for the stresses in the implant components and surrounding bones of each group. (3) Results: The implant fixture of the PEEK model exhibited the highest von Mises stress (VMS). The lowest VMS was observed in the abutment screw of the titanium model. Both implant systems demonstrated similar stress distributions and magnitudes in cortical and cancellous bones. (4) Conclusion: PEEK abutments show a similar stress distribution in the surrounding bone compared to titanium. However, PEEK absorbs the stresses within the implant system and exhibits the highest VMS values due to its low mechanical and physical properties. Therefore, PEEK abutments need improved mechanical properties for better clinical application.

## 1. Introduction

Dental implants have been widely used for restoring partially edentulous and edentulous areas since Branemark discovered osseointegration in dental implants. Dental implants are currently recognized as the optimal treatment option for replacing missing teeth [1].

Thanks to technological advancements, the success rate of implants has steadily improved, yet implant failures still occasionally occur. Factors influencing implant failure include the following: host-related factors, such as age, sex, systemic diseases, smoking, and oral hygiene status; site-related factors, such as location within the jaw, bone quality, quantity, and the presence of infection; surgery-related factors, such as initial stability, implant placement location and orientation, and surgeon skill; implant-related factors, including macro and microstructure, surface characteristics, length, and diameter; restoration-related factors, such as prosthesis type and maintenance method (screw or cement type), and occlusal scheme [2,3].

Finite element analysis (FEA) is a computational modeling technique that allows for the simulation and analysis of complex structures and materials under various conditions. It has been widely used in medicine and dentistry for decades to evaluate biomechanical behavior, predict structural responses, and optimize designs. FEA is particularly valuable in dental research as it enables detailed visualization of the stress distribution in and around implants without necessitating invasive procedures. Using FEA, researchers can simulate various loading conditions, material properties, and geometric configurations to understand the influence of these factors on implant performance and bone–implant interactions.

Among these, excessive stress and improper stress distribution transmitted to the bone around the implant pose a significant concern for bone resorption, making research in this area a highly relevant field of interest. Bone resorption around dental implants can be classified as early bone resorption, occurring shortly after implant placement, and progressive bone resorption, continuing over time [4].

The causes of early bone resorption after implant surgery include damage to surrounding tissues and the supporting bone during surgery or inappropriate forces applied to the implant during healing [4]. Additionally, progressive bone resorption leading to implant osseointegration failure is often attributed to excessive external forces post-implantation, with long-term peri-implantitis contributing as an additional factor [5,6,7,8,9]. Beyond these factors, several studies suggest that biomechanical issues could also contribute to implant bone resorption [10]. Biomechanical factors encompass implant surface shape and form, bone quality, and patient-specific elements such as bone volume. Therefore, various factors, including load position, direction, external load transmitted to the supporting bone, implant–bone interface conditions, implant diameter and length, implant thread design, and surface structure, contribute to the long-term success of implant-supported restorations [11,12,13,14].

The choice of abutment material used for implant procedures can influence the stress distribution in implant systems and the supporting bone [15]. The success of implants in edentulous areas is determined by implant form, surgical approach, healing time, and the initial load applied to the bone during the early stages of restoration [16]. Therefore, the appropriate distribution of biomechanical stresses in the supporting bone is crucial for the long-term success of implant restorations.

Polyetheretherketone (PEEK) has been introduced as an alternative to titanium abutments. PEEK, first commercially used in 1998 as a thermoplastic polymer with outstanding properties, offers excellent machinability and esthetics [17]. It has been reported as a material in orthopedic surgery to replace titanium. PEEK is a thermoplastic material, lighter than metal materials and more efficiently machinable than robust metal materials. Pure PEEK has weak mechanical properties; hence, it is often reinforced with glass or carbon fiber to enhance its mechanical and physical characteristics [17]. Previous studies evaluated stress distribution during load transmission in reinforced PEEK and compared it to titanium abutments with 30% added carbon fiber [18]. Schwitalla et al. used finite element analysis for abutments with 60% added carbon fiber [19]. However, adding carbon fiber influenced the color of PEEK significantly, with increasing carbon fiber content resulting in a darker shade [19].

Engineering analysis is necessary to understand the stress distribution in implant systems due to the risk of damage to the implant–bone interface based on the degree of osseointegration. Finite element analysis has been used in medicine and dentistry for decades [20]. This method allows for the simulation of interactions between implants and surrounding tissues. Previous research has explored stress distribution based on load direction and constraints in implant restorations, the impact of abutment material on stress distribution, and the influence of implant structure on stress distribution [21,22,23]. However, there is insufficient research on evaluating the stress distribution in the supporting bone around implants when using pure PEEK as an abutment material, and its extensive impact on the structural elements of implant systems lacks thorough investigation. Furthermore, reinforced PEEK with added carbon fiber lacks esthetics. Therefore, this study aimed to compare the effect of titanium and PEEK abutments on implant prosthetic systems and the supporting bone using three-dimensional FEA.

## 2. Materials and Methods

### 2.1. Finite Element Model

This study used CATIA V5 software (Dassault Systèmes, Vélizy-Villacoublay, France) to design components of a single-unit implant system mimicking the mandibular first molar (e.g., fixture, abutment, abutment screw, superstructure, cortical bone, and cancellous bone). For the convenience of finite element analysis, teeth adjacent to the mandibular first molar were excluded.

**Figure 1:**
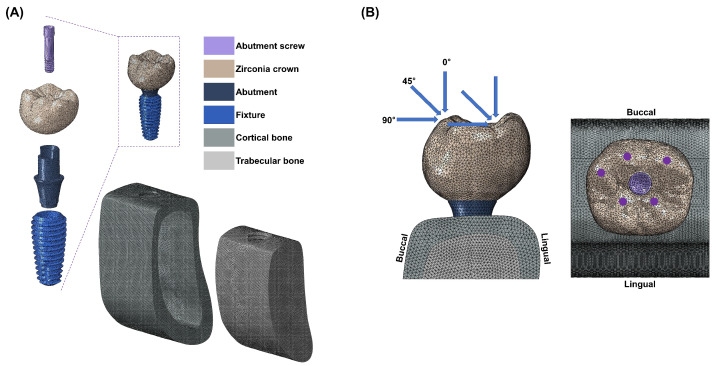
(**A**) A cross-sectional view of the three-dimensional finite element models of dental implants with the internal assembly structure and boundary and loading conditions. (**B**) External force is applied in three directions (vertical, angular, and horizontal); 60 nodes are located at 3 points on the outer inclines of the buccal cusps, and 60 nodes are located at 2 points on the inner inclines of the lingual cusps.

The implant system design was based on the commercially available MEGAGEN implant system and a previously conducted study [24]. An implant fixture (Anyone^®^; Megagen, Daegu, Republic of Korea) with a diameter of 5 mm and a length of 11.8 mm, widely used in the mandibular molar region, was used. The abutment (ZrGen Abutment; MegaGen, Daegu, Republic of Korea) had a cuff of 1.5 mm, a diameter of 4.5 mm, and a post height of 4.5 mm. The abutment screw was designed with a diameter of 1.8 mm and a length of 5.1 mm. The overall shape was designed based on a cross-sectional image of the mandible, creating a model of the mandibular bone block. The overall design of the mandible was shaped with a height of 29.5 mm, a width of 14 mm, and a thickness ranging from 1.4 to 3.7 mm, surrounded by cortical bone and separated from the cancellous bone (Figure 1A). With this model configuration, two implant system models were created, each incorporating two different abutment materials: PEEK and titanium.

### 2.2. Boundary and Loading Conditions

All materials used in this study were assumed to be homogeneous, isotropic, and linearly elastic. The Poisson’s ratio and elastic modulus of the materials were incorporated into the 3D model (Table 1) [25,26]. The finite element (FE) model underwent discretization for division into small elements. A mesh size (mean size 250 µm) was set to minimize geometric errors. Each node was assumed to be interconnected at individual points. The analysis model of the single-unit implant system, consisting of approximately 200,000 tetra 10-node elements, was analyzed using a general-purpose analysis program (Abaqus V6.12; Dassault Systemes, Waltham, MA, USA) by applying loads and boundary conditions. External loads were applied in vertical and oblique directions to describe occlusal loading. Three load directions were used to simulate occlusal loading: vertical (0 °) direction, angular (45 °) direction, and horizontal (90 °) direction. Loads were applied at 5 different loading points on occlusal surfaces: 60 nodes located at 3 points on the outer inclines of the buccal cusps, and 60 nodes located at 2 points on the inner inclines of the lingual cusps (Figure 1B). These loading points resembled contact points during mastication. The magnitude of the load was 280 N, which simulated normal masticatory force [27]. Maximum equivalent stresses were evaluated at the abutment, abutment screw, and fixture. Maximum and minimum principal stresses were evaluated for cortical and cancellous bone.

## 3. Results

**Figure 2:**
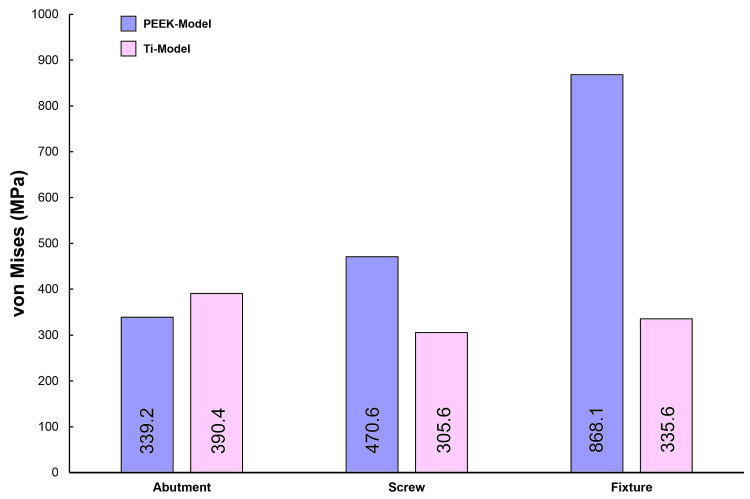
The von Mises stress values for the abutment, screw, and fixture on both models.

Figure 2 illustrates the maximum von Mises stress (VMS) values for the abutment, abutment screw, and fixture under external loading. Under external loading applications, the fixture of the PEEK-Model exhibited a higher VMS value compared to that of the Ti-Model. The lowest VMS value was observed in the abutment screw of the Ti-Model. Furthermore, the Ti-Model showed slightly higher VMS values for the abutment compared to the PEEK-Model.

**Figure 3:**
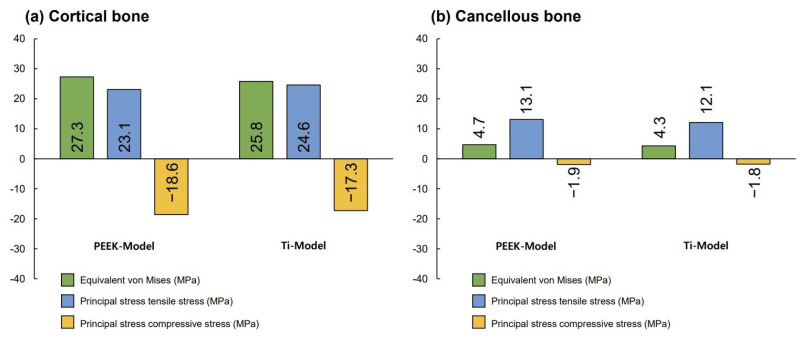
The von Mises stress and principal stress values for surrounding bones.

Figure 3 presents the VMS transmitted to the cortical and cancellous bone with the maximum/minimum principal stresses. Overall, in all FE models, the maximum and minimum stress levels were higher in the cortical bone than in the cancellous bone. Additionally, similar stress concentration points were observed in the Ti-Model and the PEEK-Model.

**Figure 4:**
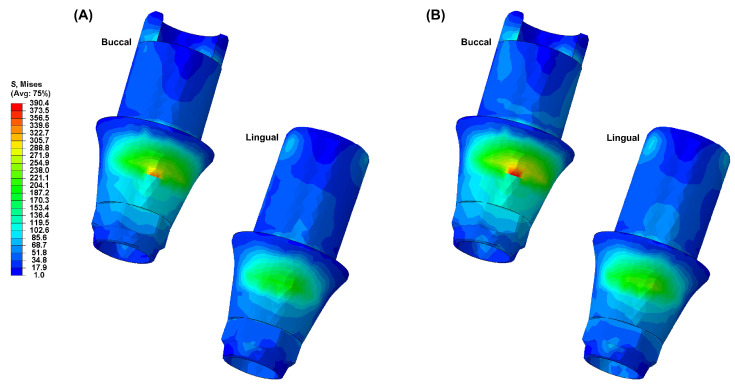
The von Mises stress distribution in the abutment: (**A**) Ti-Model; (**B**) PEEK-Model.

**Figure 5:**
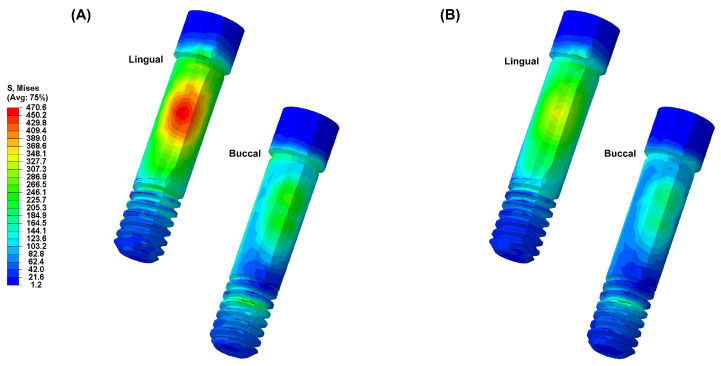
The von Mises stress distribution in the abutment screw: (**A**) Ti-Model; (**B**) PEEK-Model.

**Figure 6:**
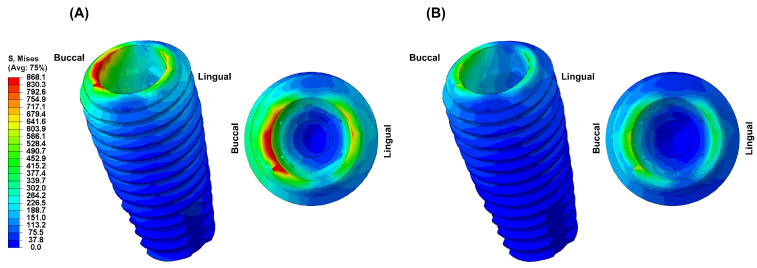
The von Mises stress distribution in the fixture: (**A**) Ti-Model; (**B**) PEEK-Model.

**Figure 7:**
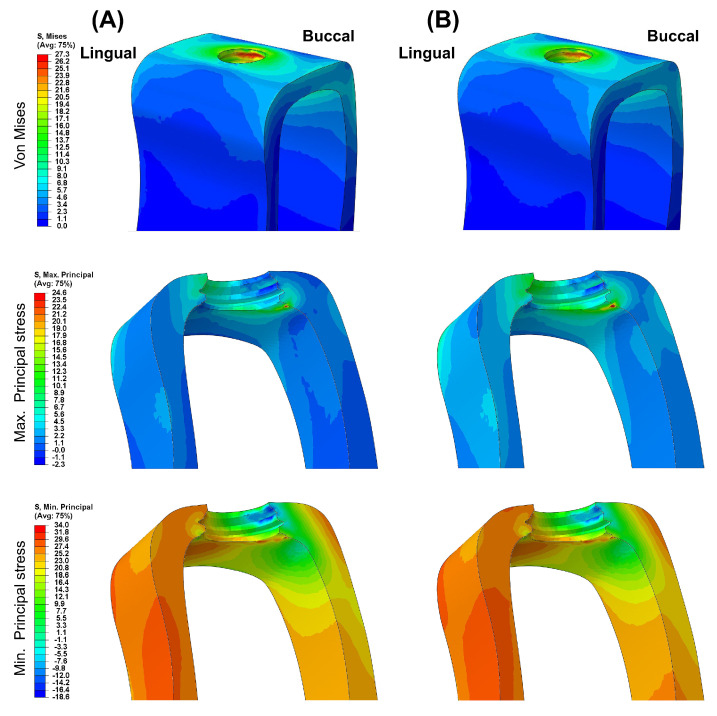
The von Mises stress and principal stress distribution in the cortical bone: (**A**) Ti-Model; (**B**) PEEK-Model.

**Figure 8:**
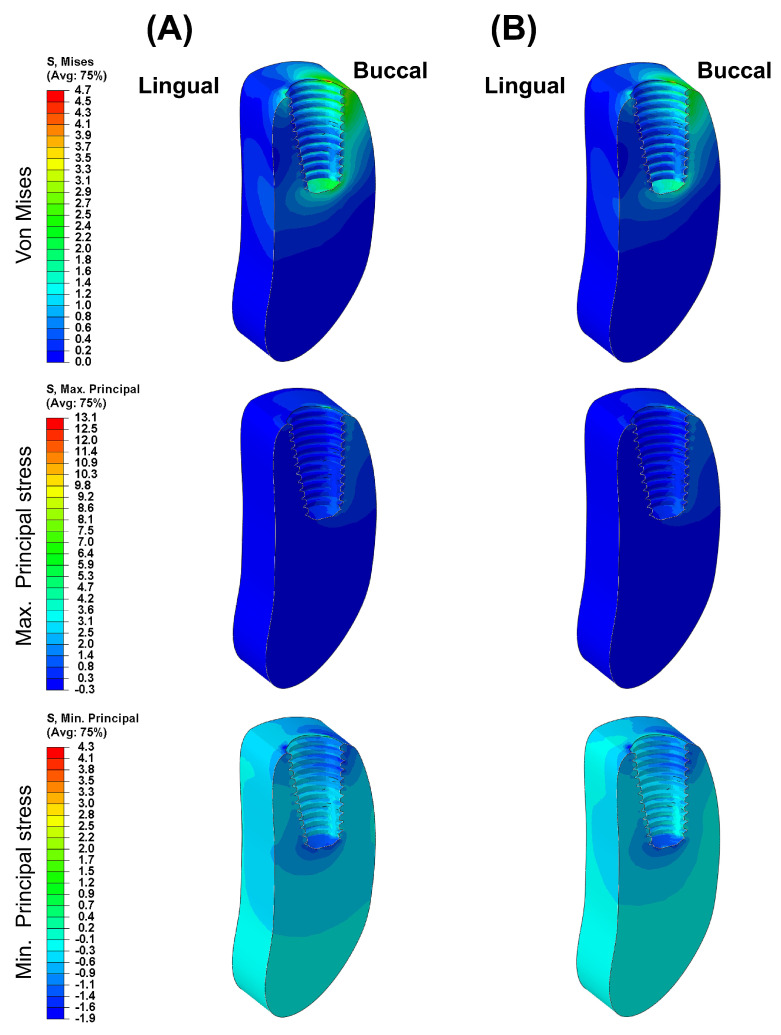
The von Mises stress and principal stress distribution in the cancellous bone: (**A**) Ti-Model; (**B**) PEEK-Model.

Figure 4, Figure 5, Figure 6, Figure 7 and Figure 8 visually present the VMS distribution in the abutment, abutment screw, fixture, cortical bone, and cancellous bone for both models. Both models showed stress concentration in the inner aspect of the fixture and the abutment base (Figure 4). The stress on the abutment screw was highest at the abutment screw body, especially in the PEEK-Model (Figure 5). The fixture exhibited stress concentration in the adjacent cervical region, especially in the PEEK-Model (Figure 6). For maximum and minimum principal stresses, stress concentration points were observed in the adjacent cortical bone (Figure 7) and the apical region of the cancellous bone (Figure 8).

## 4. Discussion

This study aimed to compare the stability of implant systems using PEEK abutments with those using titanium abutments through three-dimensional finite element analysis. This analysis allowed for a qualitative evaluation of biomechanical behaviors occurring in the oral cavity based on complex structures and various material variables, enabling an understanding of stress distribution tendencies [28].

To enhance the reliability of finite element analysis in this study, the materials applied were homogeneous and isotropic, with material properties assumed to be consistent in all directions and only two independent variables (elastic modulus and Poisson’s ratio) defined for simulation. Three-dimensional finite element analysis incorporating ideal implant designs and surrounding bone can yield successful results. For ideal modeling, element sizes should be set between 150 and 300 µm. Element sizes larger than 300 µm may produce inaccurate results, while reducing element sizes to less than 150 µm may significantly increase analysis time, reducing efficiency [29]. Therefore, this study used an average element size of 250 µm. The mechanical and physical properties of the FE model were based on validated data from previous studies. However, despite these efforts, the detailed aspects of the models and the numerical analysis of biomechanics could not encompass all biochemical ranges within the oral cavity [22,25,27]. Nonetheless, the results obtained through finite element analysis could predict the stability and efficacy of implant systems, aiding in the improvement in new designs and products [26,30].

Minimizing and evenly distributing stress transmitted to the supporting bone from loaded implant structures are essential for the long-term success of implant systems [27,31]. The experimental conditions of this study were designed to simulate an ideally treated clinical prosthetic situation. Therefore, this study used external loads in three different load directions at five points: 60 nodes located at three cusps on the buccal cusps and 60 nodes located at two cusps on the lingual cusps. Thus, external loads were appropriately distributed.

The von Mises stress is primarily used to evaluate the yield condition of isotropic and ductile metals under complex loading conditions, while the principal stress refers to the maximum and minimum stress values at a specific point in brittle solid materials when subjected to force [32]. Therefore, in this study, the von Mises stress was measured for the abutment, abutment screw, and fixture, whereas maximum and minimum principal stresses were measured for the supporting bone.

In this study, the PEEK abutment material demonstrated similar stress distributions and magnitudes in the supporting bone compared to the conventional titanium abutment implant system. However, the stress generated in the fixture of the PEEK-Model was 868.1 MPa, which was 159% higher than that of the Ti-Model. This result corresponds to a stress level similar to the yield strength of titanium material, which is 880 MPa [33]. Moreover, stress concentration on the abutment screw was also higher in the PEEK-Model than in the Ti-Model. This interpretation arises from the inherent properties of the PEEK abutment material, which has a lower modulus of elasticity than titanium, resulting in stress distribution differences between the two materials during load transmission [23,25,30]. Sarot et al. reported that implant systems using PEEK and CRF-PEEK abutments showed similar stress distributions in cortical and cancellous bone to those of titanium implant systems, consistent with our findings [18]. Finite element analysis studies applying PEEK abutments have shown that titanium abutments exhibit higher maximum equivalent stresses than PEEK abutments, which aligns with our results [18,19]. While the implant system with PEEK abutments demonstrated similar stress distributions and magnitudes in the supporting bone to those of the titanium implant system, the high stress generated in the fixture suggests a potential loss of supporting bone at the implant boundary.

Therefore, the mechanical properties of PEEK abutments need improvements to match or slightly exceed those of titanium abutments. Also, there has been no studies reporting the successful long-term prognosis of implant prosthesis using PEEK abutments. Therefore, follow-up studies are needed to demonstrate that implant systems with PEEK abutments are a viable clinical alternative to titanium implant systems.

This study on stress distribution in dental implant systems using PEEK and titanium abutments has several limitations that need consideration when interpreting the results. The present study assumes that all materials involved, including the bone and implant components, are homogeneous, isotropic, and linearly elastic. This assumption simplifies the complex nature of bone, which is heterogeneous and anisotropic, with properties that vary depending on location and density. Such simplifications may impact the accuracy of finite element analysis in predicting stress distribution. Furthermore, the finite element model used in this study simplifies the anatomical structure of the mandible. By creating a model based on a cross-sectional image, this study might not fully capture individual variations in bone density and trabecular patterns seen in actual patients. The exclusion of adjacent teeth in the model is another limitation, as these teeth can significantly influence load distribution and stress transfer during occlusal loading. Without considering the impact of adjacent teeth, the model may not accurately assess the stress experienced by the implant system. This study’s loading conditions also present limitations. Although loads are applied in three directions to simulate occlusal forces, the model does not account for the dynamic or cyclic nature of real-life mastication. In practice, masticatory forces are more complex, involving multiple directions and varying magnitudes over time, which could affect stress distribution and implant stability. Additionally, the focus on single-molar zirconia prosthetic restorations limits the scope of this study. Since different prosthetic materials possess varying mechanical properties that can influence stress distribution, examining a range of materials would offer a more comprehensive understanding of their effects on implant systems with different abutment materials.

The mechanical properties of PEEK abutments, which result in higher stress in the fixture compared to titanium, highlight the need for improvements to make PEEK suitable for clinical applications. However, the present study does not explore the limitations of current PEEK materials or the potential for developing new materials with enhanced properties. Moreover, the lack of long-term clinical data is a significant constraint. While finite element analysis predicts stress distribution, it does not replace the need for clinical studies to validate these findings and assess the long-term viability of PEEK abutments in practice.

## 5. Conclusions

In this study, the stress distribution of the surrounding supporting bone in implant systems with titanium and PEEK abutments was compared and evaluated using three-dimensional finite element analysis. The following conclusions were drawn:

1. Due to the low elastic modulus of the PEEK abutment, high VMS values were observed in the implant fixture.
2. Based on the results of this study, a PEEK abutment requires improved mechanical and physical properties for clinical application, and its clinical use is limited.
3. Different abutment materials did not significantly affect the stress distribution and magnitude in the bone around the implant.

## Figures and Tables

**Table 1:** Mechanical properties of all simulated materials.

Material	Young’s Modulus (MPa)	Poisson’s Ratio	Reference
Titanium implant	110,000	0.35	[24]
PEEK abutment	4100	0.40	[19]
Titanium abutment	110,000	0.50	[24]
Abutment screw	110,000	0.35	[24]
Zirconia crown	210,000	0.26	[21]
Cortical bone	13,400	0.30	[24]
Cancellous bone	1370	0.31	[24]

## Data Availability

The data are included in the article.
